# Normative data for the Brief Cognitive Screening Battery stratified by age and education

**DOI:** 10.1590/1980-57642016dn11-010008

**Published:** 2017

**Authors:** Mônica Sanches Yassuda, Henrique Salmazo da Silva, Thais Bento Lima-Silva, Meire Cachioni, Deusivania Vieira da Silva Falcão, Andrea Lopes, Samila Sathler Tavares Batistoni, Anita Liberalesso Neri

**Affiliations:** 1 Núcleo de Estudos e Pesquisas em Gerontologia (NEPEG), Escola de Artes, Ciências e Humanidades, Universidade de São Paulo (EACH-USP), São Paulo SP – Brasil.; 2 Grupo de Pesquisa em Neurociência, Linguagem e Cognição. Universidade Federal do ABC, Santo André SP – Brazil.; 3 Grupo de Neurologia Cognitiva e do Comportamento (GNCC), Departamento de Neurologia, Faculdade de Medicina, Universidade de São Paulo, São Paulo SP – Brazil.; 4 Programa de Pós-graduação em Gerontologia, Faculdades de Ciências Médicas, da Universidade Estadual de Campinas (UNICAMP), Campinas SP – Brazil.

**Keywords:** elderly, cognition, neuropsychiatric battery, sociodemographic data, normative data

## Abstract

**Introduction:**

Diagnosing neurocognitive disorders is challenging in low-educated individuals.

**Objective:**

To report normative data for the Brief Cognitive Screening Battery (BCSB) and to assess the association of age and education with performance on the BCSB in 240 community-dwelling elderly from Ermelino Matarazzo, São Paulo city.

**Methods:**

The inclusion criteria were scoring above the education-adjusted cut-off points on the Mini-Mental State Examination (MMSE) and below six points on the Geriatric Depression Scale (GDS).

**Results:**

Age was associated with performance on the Naming, Incidental Memory, Verbal Fluency, Clock Drawing Test, Delayed Recall and Recognition subtests. Education was associated with performance on Naming, Recognition, Verbal Fluency and the Clock Drawing Test.

**Conclusion:**

The normative values reported are relevant for diagnosing neurocognitive disorders in low-educated elderly.

## Abstract (pt)

**Introdução:**

O diagnóstico de transtornos neurocognitivos é desafiador na presença de baixa escolaridade.

**Objetivo:**

Relatar dados normativos para a Bateria Breve de Rastreio Cognitivo (BBRC) e avaliar a associação da idade e da escolaridade com o desempenho na BBRC, em 240 idosos residentes na comunidade em Ermelino Matarazzo, município de São Paulo.

**Métodos:**

Os critérios de inclusão foram pontuação acima de pontos de corte ajustados para escolaridade no Mini-Exame do Estado Mental (MEEM) e abaixo de seis pontos na Escala de Depressão Geriátrica (EDG).

**Resultados:**

A idade esteve associada aos subtestes Nomeação, Memória Incidental, Fluência Verbal, Teste do Desenho do Relógio, Memória Tardia e Reconhecimento. A escolaridade associou-se à Nomeação e Reconhecimento, Fluência Verbal e Teste do Desenho do Relógio.

**Conclusão:**

As normas reportadas são relevantes para o diagnóstico de transtornos neurocognitivos em idosos com baixa escolaridade.

## INTRODUCTION

Diagnosing neurocognitive disorders is challenging in low-educated individuals. Neurocognitive tests such as the Mini-Mental State Examination (MMSE) are strongly influenced by educational level^1-4^ and in low-educated elderly there can be doubt over whether low performance is due to cognitive deficit or limited education. The Cognitive and Behavioral Neurology Group of Clinicas Hospital of the University of São Paulo (GNCC HC/USP) developed the Brief Cognitive Screening Battery (BCSB)^5-7^ to meet the need for a battery of tests less influenced by educational level. The BCSB entails the naming and memorizing of 10 common black and white drawings. The instrument also includes the Verbal Fluency (VF) test – animals category, and Clock Drawing Test (CDT), applied between the third recall of drawings and delayed recall.^6,7^ Previous studies on the Figure Memory Test of the BCSB have shown little influence of education,^7^ while the CDT^8,9^ and VF test^10,11^ appear to be more influenced by education.

Although the CDT is a reliable screening instrument, it has not proven valid for screening dementia in elderly Brazilians with fewer than 4 years' education^9^ (see Aprahamian et al.^12^ for contrasting results). In a normative study, performance on the CDT correlated strongly with reading abilities and its scores needed to be categorized according to educational background.^13^ Similar data were found for the VF test in Brazilian adult and elderly populations with low educational level.^10,11^ In a recent study involving 218 healthy elderly,^11^ education predicted low performance on phonemic and semantic verbal fluency tasks. Illiterate elderly had the lowest scores compared to groups with 1-4 and 5-8 years' education, even after stratifying by age (60-69 years and ≥70 years).

With regard to the Figure Memory Test, when performance by literate and illiterate individuals was compared with the Word List sub-test from the CERAD battery, no significant difference was found between the two groups for the BCSB, but significant difference was evident for the Word List sub-test of the CERAD. The BCSB Figure Memory Test is highly accurate for diagnosing Alzheimer's disease (AD) in both illiterate subjects and individuals with a high level of education.^14-16^ BCSB performance correlates negatively with age and positively with education,^16^ highlighting the importance of devising age and education-adjusted norms for the items in the battery. Normative data can be useful in the context of neuropsychological assessment of elderly subjects.

Therefore, the objective of this study was to determine the influence of age and education on BCSB performance and produce normative data stratified by these factors based on a database of Brazilian elderly participants, without signs of dementia or depression, from a community in Ermelino Matarazzo, São Paulo city. These data are relevant for clinical practice given that no previous normative studies for the BCSB have been conducted.

## METHODS

**Participants.** This study was based on data obtained by the study "Profiles of Frailty in Brazilian Elderly" conducted by the FIBRA Network. This is a multi-center, multi-disciplinary research network which investigated the characteristics, prevalence and risk factors associated with the syndrome of frailty in Brazilian elderly. The study carried out in Ermelino Matarazzo was part of the UNICAMP center, with data collected between 2008 and 2009.

The Ermelino Matarazzo district is located in the Eastern region of São Paulo city. This is a region with a low socioeconomic level in a middle-income or developing country. According to the State Data Analysis System Foundation,^17^ the district covers an area of 8.7 km^2^, subdivided into 143 urban census sectors, and has a population density of 12,551.1 persons/km^2^. The elderly population (≥65 years) accounts for 4.5% of the district's population.

Probabilistic cluster sampling was carried out in two stages: census sector and household. The method of randomizing the primary sampling units (census sectors) followed the procedure adopted by the FIBRA UNICAMP center.^18^

The sample used in the present analysis comprised individuals who scored above the cut-offs on the MMSE adopted in the FIBRA project (17 points for illiterate subjects, 22 for 1-4 years' education, 24 for 5-8 years and 26 points for ≥9 years of education) and below the cut-off score (<6 points) on the Geriatric Depression Scale (GDS). These cut offs for the MMSE were calculated based on the mean for each education range (reported elsewhere)^2^ minus one standard deviation. Of the total sample of 384 elderly, 82 were excluded for having scored below the cut-off point on the MMSE. Of the remaining subsample of 302 cognitively healthy elderly, a further 62 were excluded for having scored above the cut-off point on the GDS. This gave a final study sample of 240 elderly.

**Protocol.** For this study, data for two blocks of questions from the FIBRA Network protocol were used. BCSB scores from Block C "mental status, self-assessment of memory and cognitive screening" were examined.^7^ The BCSB entails naming 10 common drawings (naming) and immediate recall (incidental memory). The figures are then displayed again and the subject asked to memorize them for 30 seconds for subsequent recall (Immediate Memory). The procedure is repeated again (Learning). Prior to recall, the individuals completes the VF test and CDT. After these two tests, the subject is asked to evoke the figures displayed earlier (Delayed Recall). Finally, the 10 figures are redisplayed mixed up with another 10 distractor figures and the participant asked to identify the figures displayed originally (Recognition).

The BCSB is scored from 0 to 10 on the figure memorizing stages. The VF score is based on the number of animals given by the participant in 60 seconds. The CDT was assessed according to the criteria of Shulman et al.^19^ with scores ranging from 0 to 5 points.

**Procedures.** After randomly selecting the 66 census sectors, the recruiters (community health workers and undergraduate students in Gerontology) performed the listings of the sectors and located those households with elderly dwellers. These elderly subjects were then asked to take part as volunteers in the study, receiving a card informing a date and place for data collection (a church or community center).

In the FIBRA study, the following inclusion criteria were adopted: age ≥65 years, able to understand the instructions, agreed to take part, permanent resident at the household and within the census sector. The exclusion criteria adopted were:

[1] Severe cognitive impairment suggestive of dementia;
[2] Wheel-chair use or bedridden;
[3] Severe stroke sequela, with localized weakness and/or aphasia;
[4] Parkinson's disease at advanced or unstable stage;
[5] Severe visual or auditory loss, hampering communication; and
[6]Terminal stage diseases. The inclusion and exclusion criteria were those used in the *Cardiovascular Health Study* and the *Women's Health and Aging Studies*, whose data were used to derive the frailty phenotype adopted by FIBRA.^20^

**Statistical analyses.** Descriptive and comparative analyses between the age (65-74 years and ≥75 years), and education groups (no education, 1-4 years and ≥5 years) were performed using non-parametric tests due to the non-normal distribution of the cognitive variables. Additionally, non-parametric partial correlations were performed to examine the association of the BCSB variables with age and education. Participants whose performance in any test was below the 5th percentile were not included in the analyses or normative tables for that particular test, as they were regarded as outliers. A 5% level of significance was adopted for all analyses (p<0.05). The statistical software program SPSS 17.0, *Complex Samples* module, was used for statistical analysis.

## RESULTS

The sample (n=240) comprised predominantly individuals aged 65-69 years (M=69; SD=2.84) who were women (62%) (Table 1). Most participants had an educational level of 0-4 years (M=3.25; SD=1.02) and family income of 1-3 minimum wages (53.8%; M=3.4; SD=3.1).

**Table 1:** Age, education, and sex for the 240 healthy Brazilian older adults.

		Mean	SD	Median	Minimum	Maximum	MMSE	n
Age (y)	65-74	69	2.84	69	65	74	25.70 (2.33)	176
	≥75	79	3	79	75	90	23.94 (3.93)	64
Education (y)	0	0	0	0	0	0	22.35 (3.11)	37
	1-4	3.25	1.02	4	1	4	25.50 (2.18)	161
	≥5	7.51	2.91	7	5	16	26.70 (1.73)	42
Sex	Women	62%					25 (3)	148
	Men	38%					26 (3)	92

The normative data stratified by age and education are given in Tables 2 and 3. Comparison between age groups showed that elderly aged ≥75 years performed worse on the following domains: Naming, Incidental Memory, Verbal Fluency, Clock Drawing Test, Delayed Recall and Recognition (Table 2). Comparison among educational levels revealed that lower-educated elderly (0 years and 1-4 years) had poorer performance than those with ≥5 years' education on the following tests: Naming, Recognition, Verbal Fluency and Clock Drawing Test (Table 3). Taken together, these findings suggest that the battery is more susceptible to the influence of age than education (Table 4). Results consistent with the above analyses were found on the correlation tests (Table 5).

**Table 2:** Scores on the Brief Cognitive Screening Battery, stratified by age and education (N=240).

	65-74 years				≥75 years				65-74 years				≥75 years		
	0 n=23	1-4 n=119	≥5 n=34		0 n=14	1-4 n=42	≥5 n=8		0 n=23	1-4 n=119	5-+ n=34		0 n=14	1-4 n=42	≥5 n=8
**Naming**								**Verbal Fluency**							
Mean	9.95	10	10		9.83	10	10	Mean	11.23	13.28	14.47		8.77	13.09	14
Median	10	10	10		10	10	10	Median	11	13	14.50		9	12	13
SD	0.22	0	0		0.39	0	0	SD	3.41	2.81	2.85		2.92	2.61	3.66
Minimum	9	10	10		9	10	10	Minimum	5	9	10		5	9	10
Maximum	10	10	10		10	10	10	Maximum	19	21	20		12	20	20
**Incidental Memory**								**Clock Drawing Test**							
Mean	6.18	6.25	6.31		6	5.97	5.60	Mean	2.50	3.26	3.85		2.20	2.88	4.14
Median	6	6.25	6		6	6	5	Median	2.50	3	4		2	3	4
SD	1.33	1.11	1.53		1.05	0.89	0.97	SD	1.42	1.37	1.23		1.10	1.47	1.07
Minimum	4	4	5		4	5	5	Minimum	1	1	1		1	1	2
Maximum	9	10	9		9	9	7	Maximum	5	5	5		4	5	5
**Immediate Memory**								**Delayed Recall**							
Mean	7.73	8.31	8.16		8.23	7.90	8.20	Mean	8.04	8.21	8.35		7.33	7.66	7.33
Median	8	8	8		8	8	9	Median	8	8	8		7.50	8	7
SD	1.32	0.99	0.88		0.93	0.88	1.10	SD	1.72	1.24	1.232		1.56	0.88	1.03
Minimum	5	7	7		7	7	7	Minimum	4	5	6		4	6	6
Maximum	10	10	10		10	9	9	Maximum	10	10	10		10	10	9
**Learning**								**Recognition**							
Mean	8.52	8.83	9.06		8.25	8.35	8.29	Mean	9.71	9.69	9.94		9.42	9.37	9.83
Median	8	9	9		8	8	8	Median	10	10	10		9.50	10	10
SD	1.12	0.94	0.83		1.36	1.03	0.49	SD	0.56	0.57	0.24		0.67	0.80	0.41
Minimum	6	7	8		6	7	8	Minimum	8	8	9		8	8	9
Maximum	10	10	10		10	10	9	Maximum	10	10	10		10	10	10

**Table 3:** Scores on the Brief Cognitive Screening Battery stratified by education (N=240).

	0 n=37	1-4 n=161	≥5 n=42	p value		0 n=37	1-4 n=161	≥5 n=42	p value*
**Naming**			0.000		**Verbal Fluency**			<0.001	
Mean	9.91	10	10		Mean	10.31	13.25	14.38	
Median	10	10	10		Median	11	13	14	
SD	0.29	0	0		SD	3.41	2.75	2.98	
Minimum	9	10	10		Minimum	5	9	10	
Maximum	10	10	10		Maximum	19	21	20	
**Incidental Memory**				0.846	**Clock Drawing Test**				<0.001
Mean	6.11	6.18	6.22		Mean	2.43	3.16	3.90	
Median	6	6	6		Median	2	3	4	
SD	1.39	1.10	0.984		SD	1.34	1.41	1.19	
Minimum	4	4	5		Minimum	1	1	1	
Maximum	9	10	9		Maximum	5	5	5	
**Immediate Memory**				0.498	**Delayed Recall**				0.668
Mean	7.91	8.22	8.16		Mean	7.80	8.07	8.205	
Median	8	8	8		Median	8	8	8	
SD	1.20	0.98	0.90		SD	1.68	118	1.24	
Minimum	5	7	7		Minimum	4	5	6	
Maximum	10	10	10		Maximum	10	10	10	
**Learning**				0.1913	**Recognition**				0.009
Mean	8.42	8.71	8.93		Mean	9.61	9.61	93	
Median	8	9	9		Median	10	10	10	
SD	120	0.98	0.83		SD	0.61	0.65	027	
Minimum	6	7	8		Minimum	8	8	9	
Maximum	10	10	10		Maximum	10	10	10	

* p values refer to the Kruskal-Wallis Test.

**Table 4:** Scores on the Brief Cognitive Screening Battery stratified by age (N=240).

	65-74 years	≥75 years	p-value*		65-74 years	≥75 years	p-value
**Naming**			0.098	**Verbal Fluency**			0.056
Mean	9.99	9.96		Mean	13.24	12.21	
Median	10	10		Median	13	12	
SD	0.08	0.419		SD	3.03	3.39	
Minimum	9	9		Minimum	5	5	
Maximum	10	10		Maximum	21	20	
**Incidental Memory**			0.0685	**Clock Drawing Test**			0.1633
Mean	6.25	5.94		Mean	3.30	2.98	
Median	6	6		Median	3	3	
SD	1.11	1.16		SD	139	1.46	
Minimum	4	4		Minimum	11		
Maximum	10	9		Maximum	5	5	
**Immediate Memory**			0.255	**Delayed Recall**			<0.001
Mean	8.20	8.02		Mean	8.22	7.55	
Median	8	8		Median	8	8	
SD	1.03	0.91		SD	1.30	1.06	
Minimum	5	7		Minimum	4	4	
Maximum	10	10		Maximum	10	10	
**Learning**			0.001	**Recognition**			<0.001
Mean	8.84	8.32		Mean	9.75	9.42	
Median	9	8		Median	10	10	
SD	0.95	1.05		SD	0.52	0.75	
Minimum	6	6		Minimum	8	8	
Maximum	10	10		Maximum	10	10	

* p values refer to the Mann-Whitney U test.

**Table 5:** Correlations of Brief Cognitive Screening Battery score with age and education.

	Naming	Incidental memory	Immediate memory	Learning test	Verbal fluency	Clock-drawing	Delayed recall test	Recognition
Age	-0.085	-0.172*	-0.182**	-0.200**	-0.177**	-0.050	-0.231**	-0.203**
Education	0.178**	0.005	0.060	0.0919	0.313**	0.302**	0.029	0.2165*

Spearman bi-variate correlations

* p>0.05

** p>0.01

## DISCUSSION

The objective of the present study was to assess the influence of age and education on the BCSB in community-dwelling elderly and to produce normative data for the battery. The results showed that the battery was influenced more by age than education. It is noteworthy that the delayed recall test was not influenced by education and can be used to identify memory impairments in elderly with different educational backgrounds. The results also confirmed that the VF test and CDT were influenced significantly by age and education.

Previous data suggest that the BCSB can effectively discriminate AD patients with different educational levels.^14-16,21^ However, no normative data for Brazilian elderly are currently available. The data from the present study are drawn from a population-based study performed in a region with a low HDI and high proportion of low-educated elderly. The current normative data complement previous validation data for the BCSB and can be of great clinical utility.

In line with previous studies, elderly with low educational level (0-4 years) performed worse on the VF test and CDT.^8-11,22^ On the Figure Memory Test, the sub-items of naming and recognition differentiated the education groups, with worse performance observed in the illiterate group. These differences in naming might be explained by previous evidence that illiterate subjects have difficulties processing two-dimensional black and white images.^23^ The figures used in the BCSB depict concrete common objects in the Brazilian context, but future studies could examine participants' performance on each individual figure to identify possible difficulties in naming or interpreting the drawings.

In summary, the results reported suggest that performance on the BCSB is significantly influenced by sociodemographic variables, namely, age and education. However, it is noteworthy that education had no impact on the delayed recall subtest of the BCSB. These data suggest that clinical interpretation of results on the subtests of this battery, frequently used for screening dementia, should take into account the sociodemographic characteristics of the patient. Normative data stratified by age and education can facilitate the identification of cognitive impairments in elderly with different educational backgrounds.
